# Interventions to improve mental well-being and sleep in paramedics: A scoping review

**DOI:** 10.1371/journal.pone.0344377

**Published:** 2026-03-09

**Authors:** Sian E. Wanstall, Brandon W. J. Brown, Meagan E. Crowther, Claire Dunbar, Robert J. Adams, Anjum Naweed, Amy C. Reynolds

**Affiliations:** 1 Flinders Health and Medical Research Institute (Sleep Health), Flinders University, Bedford Park, South Australia, Australia; 2 Monash University, School of Psychological Sciences, Notting Hill, Victoria, Australia; 3 AISH Sleep Health Clinics, Flinders University, Bedford Park, South Australia, Australia; 4 Central Queensland University, Appleton Institute for Behavioural Science, Wayville, South Australia, Australia; Khyber Medical University, PAKISTAN

## Abstract

**Background:**

Paramedics face unique occupational hazards, including high operational demands, trauma exposure, and shift work, all of which impact mental well-being. Suboptimal sleep is also common in this workforce and closely linked to adverse mental health outcomes. This scoping review synthesizes evidence to date on interventions to support paramedic mental well-being including sleep-based interventions.

**Materials and methods:**

This review was pre-registered on the Open Science Framework (https://doi.org/10.17605/OSF.IO/7VSD9). Systematic database searches were conducted in October 2024 for original research published after 2004. Data were narratively synthesised, and findings reported following established guidelines.

**Results:**

Nineteen sources were included, involving 1,067 participants across seven countries. Seventeen interventions were examined, predominantly via randomized controlled trials (58%), utilizing a total of 43 different measurement scales to evaluate mental health and sleep outcomes. Interventions included psychological (37%), sleep, fatigue and/or shift work (32%), and complementary and alternative medicine (32%) approaches which primarily focussed on the individual-level (94%). Studies were limited by sample sizes, design and quality, limited long term follow-up, and low baseline symptoms.

**Conclusions:**

This review highlights a critical gap in robust, evidence-based, system-level interventions to address poor sleep and mental well-being in paramedics. Future research should prioritise co-designed, context-sensitive approaches, ideally integrated within organisational structures to ensure relevance and accessibility.

## Introduction

Paramedics deliver a diverse range of essential health care in the out-of-hospital setting and are an integral component of contemporary healthcare systems. [1,2] The paramedic role is a unique and challenging modern healthcare role, encompassing routine exposure to acute and chronic occupational hazards, which have negative associations with paramedic well-being. [3–5] In Australia, these associations include poor psychological well-being, with paramedics reporting mental health conditions at eight times the rate of all other occupations. [6] Symptoms of anxiety, depression, and post-traumatic stress disorder (PTSD) are prevalent among paramedics, and experienced at significantly higher rates compared to the general population [7].

In a 2020 survey of Australian paramedics (*n* = 184), 27% reported high or very high levels of psychological distress. [8] More broadly, ambulance personnel report psychological distress at higher rates (27%) than the general population (11%). [9–11] A 2018 systematic review suggests that global prevalence of PTSD among ambulance personnel is 11%, and 15% for depression or anxiety. [11] Paramedics also experience the highest rates of non-fatal suicide attempts compared to other emergency service workers, [12] and the proportion of paramedics who die by suicide is 2.6 times higher than the general population. [13] There is strong evidence that burnout is also prevalent, with more than 50% of Australian and UK paramedics reporting symptoms of burnout, [14–16] and similar estimates reported in US emergency medical service (EMS) workers (47%). [17] Of added concern, paramedics are less likely to seek help for mental health concerns than the general working population [18].

While trauma exposure and organisational stressors are most commonly cited as primary factors contributing to poor mental well-being among paramedics, [18,19] another factor which has received less attention to date, is sleep health. Several studies demonstrate a high prevalence of poor sleep among paramedics. [7,20–23] For example, excessive daytime sleepiness, poor sleep quality, and fatigue are commonly reported in paramedics. [24–26] Short sleep duration is also common; in a large sample of American paramedics, 72% reported less than seven hours of sleep on workdays, [20] and an average of six hours was reported in another large sample of Canadian paramedics. [27] Sleep problems among paramedics are likely related to shift work exposure (work conducted outside of 8am-6 pm) [28] to some extent. [29] For example, shift work is independently associated with poor mental health, including anxiety and depression, [30] and sleep is thought to play an important role in this relationship [31–33].

Considering psychological well-being and sleep together, recent evidence also suggests that sleep disorders may play a role in the relationship between shift work and poor mental well-being. [34] Sleep disorders are common among paramedics, with Australian paramedics reporting a significantly higher prevalence of insomnia, narcolepsy, and shift work disorder than the general population, and have nearly double the risk of obstructive sleep apnoea (OSA) (41.5% and 24% respectively). [7] In a survey-based study, 60% of Canadian paramedics screened positive for insomnia, and were also 3.81 times more likely to meet screening criteria for a common mental health disorder than those without. [27] Given the link between sleep and mental health outcomes, [35] poor sleep represents an important, but largely under-explored, area for potential intervention to improve mental well-being in paramedics [36].

Despite a growing body of research into paramedic mental health broadly, targeted interventions are very limited. [37,38] Prior studies have typically focussed on broader groups, such as first responders or public safety personnel, with paramedics comprising only a small proportion of the sample. [39–45] This is problematic given that paramedics often experience poorer mental health outcomes relative to other occupational groups, and appear to have low help-seeking behaviours. [6,18] Additionally, paramedics occupy a distinctive role characterised by unique and inherently demanding stressors. They are required to make autonomous, complex decisions in dynamic, time-pressured and high-stress situations, often with high stakes for their patients. [46] Exposure to violence, traumatic events, and death, is also common, often resulting in significant emotional labour and suppression. [47] Often, these stressors are encountered in unpredictable and uncontrolled environments. [4] In some jurisdictions, paramedics are also experiencing significant organisational demands including unprecedented workload and ambulance ramping (delay in handover of an ambulance patient to definitive care), [48] which contribute to late or no meal breaks, decreased on-shift downtime, and limited time to debrief challenging cases. Poor workplace culture, high levels of bullying, and poor organisational support can also be prevalent. [49,50] Additionally, due to the requirement to provide 24/7 out-of-hospital care, paramedics experience these challenges whilst largely engaging in shift work.

While reviews into interventions for paramedic mental well-being exist, these have been limited by population of interest, [39,40,42,45] geographic location, [38,45] scope of interventions or outcomes, [39,40,42] and mental well-being or sleep outcomes investigated secondary to occupational performance or clinical safety endpoints. [51,52] Given this context, the purpose of the current scoping review was to conduct a global review exploring interventions explicitly aimed at supporting paramedic mental well-being with consideration for sleep-based interventions.

### Objectives

- To summarise existing literature on interventions to support paramedic mental well-being.
- To identify whether interventions targeting sleep have been considered as a meaningful way to support paramedic mental well-being.
- To synthesise existing evidence and identify any gaps in the literature that may exist.

## Materials and methods

The current scoping review protocol was registered on Open Science Framework [53] on 01/02/2024, prior to searches being conducted, DOI: 10.17605/OSF.IO/7VSD9. Existing methodological frameworks were used to develop the current scoping review, [54,55] which is reported following PRISMA-ScR guidelines. [56] A scoping review was identified as the most appropriate methodology given the paucity of prior research and the overarching aim of summarising the breadth of research that does exist [57].

### Eligibility criteria

Peer reviewed, original research articles published after 2004 and written in English were eligible for inclusion. The search date was selected to reflect the relative recency of paramedicine as a profession. The focus of this review was on original articles with intervention methodologies; consequently, reviews, case studies, and editorials/expert opinion articles, and theses were not eligible.

#### Population, concept and context.

The population of interest were paramedics. For the purpose of this review, the role title ‘paramedic’ also encompassed emergency medical technicians (EMTs), EMS workers and student paramedics. In some jurisdictions, individuals work in a dual role of firefighter/EMT. Only studies which explicitly identified participants as having an EMT role were eligible for inclusion. Similarly, for studies investigating groups labelled ‘first responder’ or similar, if no more information was given on population, the study was not eligible for inclusion as it was not possible to determine the proportion of participants who were paramedics in the study. For studies investigating a broader group encompassing paramedics, those who presented data that could be independently interpreted by occupation, or those where the majority of participants (>70%) were clearly identifiable as paramedics, were included.

The concept of interest was any intervention aimed at improving paramedic sleep health or mental well-being outcomes. Studies assessing an intervention with or without a comparator/control were considered. All components of sleep and mental well-being were considered, including but not limited to, sleep and mental health symptoms or disorders, suicidality, stress, burnout, fatigue, coping, and resilience. These outcomes could be primary or secondary endpoints, and both validated and non-validated outcomes were considered for inclusion. The broad context was operational ambulance or EMS settings or other similar contexts (e.g., hospitals), or student paramedics studying in tertiary or organisational settings. Geographic location of the research, as well as the setting in which the intervention was delivered (e.g., community, organisation, university research) were not limited.

### Information sources

Searches were conducted across four online databases for relevant published studies: MEDLINE, CINAHL Complete, PsycInfo, and Web of Science. Final searches were conducted on 28/10/2024. Searches using key words were also conducted in Google Scholar, Trove, and Amber. Backward and forward citation searching was conducted for all included studies. Backward citation searching involved manually screening the reference lists of each included article and forward citation searching conducted in Google Scholar by reviewing articles that cited each included study. Manual searching was also conducted in the following non-indexed paramedicine-specific journal websites: International Paramedic Practice and Journal of Paramedic Practice. Records identified through citation and manual searching were screened using the same process as records identified through database searching.

### Search

The search strategy was developed by the research team. Search terms were partially informed by literature outlining search terms specific to paramedic research, [58] as well as commonly used terms in key articles. After conducting preliminary searches, the search strategy was reviewed by the research team and further refined. Delays in the review process necessitated updated database searches, which were conducted with a refined search strategy. The refined search strategy involved more accurate use of syntax in the search string and inclusion of further search terms. A full summary of the search strategies used in final database searches is provided in supporting information (S1 Table).

### Study selection

One author conducted all searches. Potentially relevant sources (title and abstract) were imported into Covidence (an online software platform developed for conducting systematic reviews), where duplicates were automatically removed. Two authors independently reviewed and selected sources of evidence categorising as ‘yes’ or ‘no’ in accordance with the inclusion and exclusion criteria. Sources lacking available information in the title or abstract were retrieved for full-text review. A document retrieval service was utilised where full-text sources could not be located (*n* = 3). In studies where the sample was comprised of other occupations, but paramedics represented >70% of the sample (*n* = 5), study authors were contacted for clarification of outcomes by occupational group, if available. Disagreement regarding inclusion was addressed though discussion between the screening authors, with any ongoing disagreement discussed with a third author for final consideration.

### Data charting and synthesis

A pre-determined data charting plan was established for this review, [59] and data items were extracted in accordance with PRISMA-ScR guidelines, into a database. One author assessed full-text versions of all potentially relevant sources against the *a priori* criteria prior to data extraction. A second author independently screened randomly selected studies (*n* = 10, 9%) deemed eligible for inclusion to ensure reliability, with 100% agreement reached. Synthesis of results was conducted narratively and via summary tables/figures. Results were organised broadly by intervention type and outcome measures.

### Critical appraisal of individual sources of evidence

The Mixed Method Assessment Tool (MMAT, version 2018) was utilised as a critical appraisal tool to assess source quality. [60] Each study was assessed against the MMAT criteria relevant to its design type (qualitative, quantitative, or mixed-methods), with judgments made regarding methodological rigor, including clarity of research questions, appropriateness of data collection and analysis, and risk of bias. Scores from these assessments were used to inform the interpretation of evidence and the confidence placed in study findings. The MMAT is a widely used quality appraisal tool which facilitates reviews including studies with varying methods and designs. Quality appraisal of all included sources was conducted by one author, with randomly selected sources (*n* = 10) independently reviewed by a second author.

## Results

### Selection of sources of evidence

**Fig 1:**
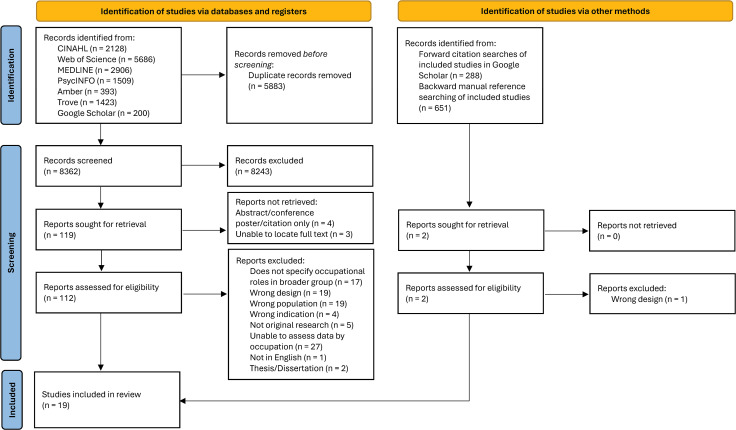
PRISMA flowchart of screening of records.

Fig 1 provides a flowchart for article selection and inclusion. In total, of the 121 records sought for retrieval, 114 were located and screened, and 19 were included in this review [61–79].

### Characteristics of sources of evidence

Table 1 provides a summary of included studies. Across the 19 studies included, there were a total of 1067 participants, with sample sizes ranging from *n* = 10 [73] to *n* = 678 [70]. Across the 16 studies reporting mean age, the pooled mean was 28.9 ± 5.7 years. Overall, 13 studies (68%) examined experienced paramedics, with a pooled mean of 8.2 ± 4.1 years of occupational experience, reported by 9 (47%) studies. The remaining six studies (32%) examined paramedic students. [61,65–67,75,79] Five (26%) of the included studies were conducted in both Iran [62,64,76–78] and the USA, [63,68–71] three (16%) in both Australia [65,72,75] and Canada, [61,66,67] and one study each in Korea, [79] Japan, [73] and Turkey [74].

**Table 1:** Characteristics of included studies.

Author (year) C*ountry*	Population (sample size) % of sample paramedic population	Aim/s Study design *Theoretical framework*	Intervention/s and delivery	Key Findings *statistically significant change
**Psychological interventions**				
Anderson et al. (2017) *Canada*	Student paramedic (*n* = 138) 100	Examine the impact of a short-duration, self-paced resiliency training course. RCT	**Online resiliency training program** A self-paced, 6–8 hour online course conducted over a 2-week period. Content included: stress physiology, trauma, workplace risks and benefits for paramedics, resiliency, stress management, and building resiliency through self-awareness, support systems, and coping strategies. Measures taken pre- and post-intervention.	↑ RS* (improved resilience)
Behnammoghadam et al. (2019) *Iran*	EMT (*n = 5*0) NR#	Evaluate the effect of EMDR on stress intensity in EMTs scoring >19 on Elkin/Alken Stress Symptom scale. RCT	**Eye Movement Desensitization and Reprocessing (EMDR)** Five, 45–90 min consecutive sessions with 8 stages: history taking, preparation, evaluation, desensitization, implementation, physical scan, completion, and re-evaluation. Participants recalled distressing events, followed eye movements, and assessed their mental discomfort and cognitive validity throughout the process.	↓ stress intensity* (reduced self-reported stress intensity)
Dearing & Kippenbrock (2022) *USA*	Paramedic (*n* = 23) 30.4	Develop a PTSD prevention and surveillance program that simultaneously educates on the dynamics of PTSD through a QI initiative. Pre-post study	**PTSD prevention, surveillance, and education program** Online and in-person education on PTSD symptom recognition, and critical incident stress and mental health services, along with anti-stigma posters, experiential learning for resilience, mindfulness exercises, and exercises to enhance coping and support. Delivered over 2 months. Measures taken pre- and 3- and 6-months post intervention.	↓ PCL-5 symptoms (reduced PTSD symptom severity) ↔ ProQOL (quality of life)
Ebrahimian et al. (2021) *Iran*	EMS technician (*n* = 64) 100	Determine the effects of psychological hotwash on resilience. RCT	**Psychological hotwash (debriefing)** Post-night shift participants completed 8 > sessions (2-hrs initial session, 70–90 minutes following sessions) covering shift events, reactions, symptom acknowledgment, coping strategies, and re-entry to life. Delivered (with assistance from psychologist) in the occupational setting over 1 month. Measures taken pre- and post-intervention.	↑ EMSRS post intervention* (improved resilience) ↔ EMSRS at 6 weeks
Pinks et al. (2021) *Australia*	Student paramedic (*n* = 78) 100	Examine if participation in a peer support group improves emotional expression and emotion-focused coping. Quasi-experimental *Cohen and Wills’ Buffering Hypothesis theory* *CARES skills framework*	**Peer support** A 90-min workshop on the CARES skills framework, followed by bi-weekly peer support group meetings. Participants engaged in mindfulness exercises, shared personal experiences, and provided feedback using reflective listening and empathy. Delivered over 12 weeks. Measures taken pre- and post-intervention.	↔ DASS-21 (depression) ↔ GHSQ (help-seeking) ↑ EACS*(improved emotional approach and coping) ↑ EES* (improved emotional expressivity) ↔ MPSS (perceived social support)
Porter & Johnson (2008) *Canada*	Student paramedic (n = 23) 100	Examine whether participation in a psychoeducational group intervention impacts pre-examined predictors of psychological distress. RCT *Cognitive-behavioural theory of change*	**Psychoeducation** A 13-session group counselling intervention focusing on fostering peer support, positive attitudes towards emotional expression, and adaptive coping strategies. Delivered over 4 months. Measures taken pre- and 6-months post-intervention.	↔ SCL-90-R, (psychological symptoms) MBI (burnout), WOC (coping), AEE (attitude toward emotional expression), perceived peer support (according to the Peer Support Crisis Questionnaire)
Vaughan et al. (2020) *Canada*	Student paramedic (n = 34) 100	Investigate the initial impact of an online resiliency training program on resilience and explore the potential skill decay. Pre-post study	**Online resiliency training program** See Anderson et al., (2017), same intervention. Measures taken pre- and 3-, 6-, and 9-months post-intervention. FU timepoint randomly assigned.	↔ RSA post intervention (resilience) ↓ RSA at 6 months* ↓ RSA at 9 months*
**Sleep, fatigue & shift work interventions**				
Patterson et al. (2015) *USA*	EMT/paramedic (*n* = 100) 9/65	Determine if a text-message based program reduces perceived sleepiness, fatigue, or difficulty with concentration during shift work, or if it has an impact on established sleep health and fatigue indicators. RCT	**Real-time text-message-based fatigue support** Fatigue reduction and alertness enhancement through sending targeted text messages linking sleepiness and fatigue to negative safety outcomes. Four mitigation strategies recommended (napping, caffeine, exercising, conversation). Subsequent tracking of effectiveness based on participant responses. 90-day study period.	↔ ESS (sleepiness) ↓ PSQI* (sleep quality) ↔ CFQ (fatigue) ↔ OFER (fatigue) ↑ SFAB (1 of 8 domains only – improvement in attitudes toward improving alertness on shift) ↔ SAS (attitudes about shift scheduling)
Patterson et al. (2024) *USA*	EMT/paramedic (*n* = 26) 85.7	Test the effect of a 30-min vs 2-hr nap opportunity taken during a simulated night shift on cognitive performance, fatigue, sleepiness, mood states, and sleep at the end of shift and throughout post-night shift recovery opportunity. Randomised crossover trial	**Scheduled napping** Thirty min nap vs 2-hr nap vs no nap (control) at 2am during a 12-hr in-lab night shift within a 72-hr study period. Night shift finishes at 7am. Following night shift, is a 6-hr post-shift recovery period without sleep (outcomes measured here), finishing at 1 pm. After post-shift recovery, 2-hr nap 1 pm-3 pm (EEG measured). Participant went home at 5 pm with actigraphy + prescribed sleep opportunity 10 pm-7 am.	After a 2-hr vs 30-min or 0-min nap (during shift): *Post-shift recovery (7am-1 pm):* ↓ SR sleepiness, fatigue, difficulty concentrating and exhaustion* ↓ PVT-B lapses (8am only*) ↔ in other measures of mood *Beyond post shift recovery:* ↔ objective sleep measures (actigraphy, EEG) in subsequent naps and sleep following sleep episodes
Patterson et al. (2023)a *USA*	EMT-Basic/ paramedic (*n* = 678) 94.4 (Intervention) 93.7 (control)	Determine if providing education and training on the importance of sleep health and dangers of fatigue impacts key indicators of sleep quality and fatigue. Cluster RCT	**Sleep health and fatigue education** Ten modules on sleep health and fatigue management tailored for EMS workers including evidence summaries, quizzes, and expert interviews. Three month delivery period and 6-month total duration for WL control cross-over. BL, 3- and 6-month FU.	↔ PSQI (sleep quality) ↔ CFQ (fatigue) ↔ ESS (sleepiness) ↔ OFER (fatigue) ↔ EMS-SAQ (safety attitudes of EMS workers) ↔ SAS (attitudes about shift scheduling)
Patterson et al. (2023)b *USA*	EMT/paramedic (n = 26) 85.7	Test the effect of different nap durations on post-nap cognitive performance and alertness. Randomised crossover trial	**Scheduled napping** See Patterson et al. (2024), same study. PVT-B (as a measure of sleep inertia) also assessed at start/end of simulated night shift, pre-nap, and post-nap at 0, 10, 20 and 30 mins. At same time-points: SR fatigue, sleepiness, alertness, and difficulty with concentration measured.	*At 0-min post-nap:* ↑ lapses and reaction time for both 30-min and 2-hr nap, ↔ between ↑ SR sleepiness and ↓ concentration and alertness for 2-hr nap ↓ alertness for 30-min nap *At 10-, 20- or 30-min post nap:* ↔ for all PVT-B outcomes for both nap conditions ↔ for sleepiness, fatigue and mood for both nap conditions
Shriane et al. (2024) *Australia*	Paramedic (*n* = 58) 100	Trial a sleep health-based mobile health intervention, incorporating sleep hygiene advice tailored for shift workers. RCT	**Mobile health application** The ‘ Sleepfit’ app was used to assess and improve sleep health through tailored and relevant fatigue management advice and educational content. Fifty-two activities within 10 modules (i.e., quizzes, guided meditations) with optional engagement features (i.e., sleep diaries, daily check-ins). Measures taken pre- and post-intervention (14 days), with 3-month FU.	↔ ISI (insomnia severity), FFS (fatigue), ESS (sleepiness) and SHI (sleep hygiene) ↓ ISI* and SHI* when waitlist control + active arm collapsed together (14 day timepoint)
Takeyama et al. (2009) *Japan*	Paramedic (n = 10) 100	Examine the effects of a modified shift system on fatigue. Quasi-experimental	**Modified night shift system** Two teams of 5, one group working a traditional shift system (T-shift, all 5 working 24hrs with no scheduled naps), the other 5 in a modified system. The modified system was within the traditional 24-hour shift system, but 2 participants took naps from either 9:30 pm-3am (C-shift) or from 3am-8:45am (B-shift) (randomly assigned). An extra participant (D-shift) was added to cover emergency calls during nap periods. Total 42-day field study period. Measures taken before and after night work in T-, B-, and C-shift groups.	↔ subjective fatigue ↑ SR nap time in C shift compared with T shift
**Complementary and alternative medicine interventions**				
Gündoğan & Kaplan Serin (2022) *Turkey*	EMT/paramedic (*n* = 30) 100	Investigate the effects of progressive muscle relaxation exercises on COVID-19 related fear, anxiety, and sleep quality. RCT	**Muscle relaxation training** Theoretical and practical training on progressive relaxation exercises from a physiotherapist and psychologist. Participants practiced guided breathing and muscle stretching exercises using a CD with relaxing music, 2 x daily for 5 days. Measures taken pre- and post-intervention.	↓ GAD-7 * (reduced anxiety) ↓ PSQI * (improved sleep quality) ↔ CP19-S (fear of COVID-19)
Hill et al. (2021) *Australia*	Student paramedic (n = 89) 100	Examine whether the presence of a wellness dog had any impact on SR emotional well-being. Pre-post study	**Wellness dog** A 1-year-old Labrador bred for service work attended university classes every other week for 15 mins (10 mins prior to class, until 5 mins into class). Eligibility included participants who attended class on 2 days the dog was present, and 2 days the dog was absent. Delivered over one academic semester. Measures taken at the beginning of each class over the semester.	↑ BEES scores (improved emotional wellbeing)
Jadidi et al. (2023) *Iran*	EMT (*n* = 60) 100	Investigate the effect of stachys lavandulifolia (medicinal plant with potential anxiolytic effects) on occupational stress. RCT	**Stachys lavandulifolia tea** Participants consumed 1g of stachys lavandulifolia tea in 200ccs of boiling water 2 x daily for 2 months. Control group participants consumed ordinary black tea at the same frequency and duration. Measures taken pre- and post-intervention.	↓ HSS-35* (reduced stress)
Mahdizadeh et al. (2019) *Iran*	EMT (*n* = 59) 78	Determine the effect of massage therapy on occupational stress. RCT	**Swedish Massage** Participants received a full body massage for 60–70 mins, 2 x weekly, with 1–2 day intervals between sessions dependent on shifts. Intervention delivered by the researcher at the end of shift in the participants’ workplace over a 4 week period. Measures taken pre- and post-intervention.	Not available
Shabani et al. (2023) *Iran*	EMS worker (*n* = 69) 100	Investigate the impact of two non-pharmacological interventions (foot reflexology with olive oil and aromatherapy with peppermint oil) on fatigue and stress. RCT	**Foot reflexology with olive oil & peppermint essential oil inhalation** Participants in one group received the foot reflexology (delivered by researcher) for 3 x 40-min sessions a week. Participants another group inhaled 3 drops of 10% peppermint oil on a gauze pad for 5 mins. Three-month study period. Measures taken pre- and one hour post-final intervention.	↓ OSIPOW foot reflexology* and peppermint oil* (reduced occupational stress) ↓ FSS foot reflexology* and peppermint oil* (reduced fatigue severity) ↔ between groups.
Yoo et al. (2023) *Korea*	Student paramedic (n = 55) 100	Investigate the effects of meditation on depression, anxiety, stress, and sleep quality. Quasi-experimental	**Meditation** An 8-week course, held 3 x weekly for 50 mins. Content focused on the ‘True Self’ meditation method to eliminate negative mindsets and false perceptions through self-reflection and the ‘mind subtraction method’. Each session included a short lecture and meditation practice, guiding participants through progressive levels. Measures taken one week pre- and one week post-intervention.	↔ K-CES-D (depression) ↓ BAI* (reduced anxiety) ↓ SRI* (reduced stress) ↑ LSEQ* (improved sleep quality)

^#^All EMTs in Iran are reportedly Male, sample likely 100% male (Ebrahimian et al., [2021]), ^Authors describe scale as Alken/Elkin interchangeably, BL = baseline, FU = follow up, NR = not reported, SR = self-reported, WL = waitlist.

Intervention abbreviations: CARES = Connect to emotion, Attention training, Reflective listening, Empathy, and Support help seeking, EMDR = Eye Movement Desensitization Reprocessing, EMS = emergency medical service, EMT = emergency medical technician, PTSD = Post Traumatic Stress Disorder, QI = Quality Improvement, RCT = randomised control trial.

Measure abbreviations: AEE = Attitude Towards Emotional Expression Scale, BAI = Beck Anxiety Inventory, BEES = Brief Emotional Experience Scale, CFQ = Chalder Fatigue Questionnaire, CP19-S = Fear of COVID-19 Scale, DASS-21 = Depression, Anxiety and Stress Scale 21, EACS = Emotional Approach Coping Scale, EEG = Electroencephalogram, EES = Emotional Expressivity Scale, EMSRS = Emergency Medical Services Resilience Scale, EMS-SAQ = Emergency Medical Services Safety Attitudes Questionnaire, ENSS = Expanded Nurses Stress Scale, ESS = Epworth Sleepiness Scale, FSS = Fatigue Severity Scale, GAD-7 = Generalized Anxiety Disorder-7, GHSQ = General Help-Seeking Questionnaire, HSS-35 = Hospital Stress Scale, ISI = Insomnia Severity Index, K-CES-D = Korean version-Center for Epidemiological Studies-Depression Scale, LSEQ = Leeds Sleep Evaluation Questionnaire, MBI = Maslach Burnout Inventory, MPSS = Multidimensional scale of perceived social support, OFER = Occupational Fatigue, Exhaustion, Recovery Scale, OSIPOW = Occupational Stress Inventory, PCL-5 = Post Traumatic Stress Disorder Checklist for DSM-5, ProQOL = Professional Quality of Life, PSQI = Pittsburgh Sleep Quality Index, PVT-B = Brief Psychomotor Vigilance Test, RSA = Resilience Scale for Adults, RS = Resilience Scale, SAS = Schedule Attitudes Survey, SCL-90-R = Symptom Checklist-90-Revised, SFAB = Sleep, Fatigue, & Alertness Behaviour Survey, SHI = Sleep Hygiene Index, SRI = Stress Response Inventory, WOC = Ways of Coping Questionnaire.

### Study designs and comparators

Randomised control trials (RCT) (including one cluster RCT), were the most commonly reported study design (58%), predominantly assessing an intervention against an inactive comparator. [61,62,64,66,68,74,77,78] Two studies (11%) utilised a waitlist control, [70,72] and one used an active comparator. [76] Other study designs included randomised crossover trials (11%), [69,71] quasi-experimental studies (18%), [65,73,79] pre- post-test studies (11%), [63,67] and one mixed methods study (pre- post-test combined with qualitative focus groups) [75].

#### Outcome measures.

There was heterogeneity between outcome measures, with 43 measurement scales used across the 19 included studies. The two most commonly used sleep measures were the Pittsburgh Sleep Quality Index (PSQI) (*n* = 3 [68,70,74]), and the Epworth Sleepiness Scale (ESS) (*n* = 3 [68,70,72]). Measures can be grouped broadly as: sleep (11 measures), depression, anxiety, and stress (11 measures), resilience, coping and burnout (8 measures), attitudes and behaviours (5 measures), fatigue (4 measures), and emotion and quality of life (QOL) (4 measures). Most studies utilised validated measures, and the majority of measures across all studies were self-reported (95%). Only two studies (11%) used objective sleep measures (electroencephalogram, [69,71] actigraphy [69], Brief Psychomotor Vigilance Test [PVT‐B] [71]), and were part of the same broader study. Few studies collected follow-up measures beyond post-intervention, with the exception of Ebrahimian, [64] Patterson, [70] Shriane, [72] and Vaughan. [67] Follow up in three of these studies was limited by high attrition/low adherence. [67,70,72] All outcome measures utilised across studies are provided in supporting information (S3 Table).

#### Interventions.

Interventions also varied considerably across studies. With the exception of one study, [73] all interventions targeted the worker, with both individual and group approaches. Broadly, interventions could be grouped into three broad categories: Psychological, Sleep fatigue and shift work, and complementary and alternative medicines. These are discussed by group below.

*Psychological* (*n* = 7) intervention approaches targeted knowledge, attitudes, and skills regarding individual resilience, coping, and mindfulness, and mental health symptoms and support avenues. These interventions were delivered via various modes including online [61,63,67] and group education, [63] peer support, [65,66] and group counselling. [66] One of these interventions was aimed specifically at PTSD prevention. [63] Other interventions included post-shift debriefing (referred to as psychological hotwash) [64] and Eye Movement Desensitization Reprocessing (EMDR) treatment in EMTs self-reporting average or higher stress levels [62].

*Sleep, fatigue, and shift work* (*n* = 6) interventions included scheduled napping, [69,71] sleep health/fatigue support and education, [68,70,72] and shift schedule modification. [73] The majority of these studies were conducted by the same lead author in the USA. [68–71] The two napping studies assessed the effect of napping and different nap durations on measures of sleep, fatigue, and mood. [69,71] The three studies evaluating sleep health/fatigue education or support included a tailored sleep and fatigue education intervention, [70] a real-time text message-based fatigue support intervention, [68] and a sleep health-based mobile phone app which provided shift work tailored advice and education to support healthy sleep practices. [72] One study examined an organisational-level intervention involving a modified shift system that incorporated uninterrupted nap opportunities, and explored the impact on fatigue [73].

*Complementary and alternative medicine* (*n* = 6) interventions included muscle relaxation training to reduce COVID-19 related fear and anxiety and improve sleep quality, [74] a wellness dog to support emotional well-being, [75] anxiolytic tea [76] and Swedish massage [77] to reduce occupational stress, and meditation to reduce anxiety, and stress, and improve sleep quality. [79] One study compared two alternative therapies, foot reflexology and essential oil inhalation, evaluating their impact on measures of fatigue and occupational stress [78].

### Critical appraisal within sources of evidence

The quality of the included sources is summarised and presented in Table 2. Most studies provided clear research questions with appropriately aligned methods, measures and analyses. Only one study achieved “Yes” for all quality appraisal items. [60] Common concerns across the included RCT studies were a lack of appropriate or transparent randomisation methods and non-comparable groups at baseline, and many were either not blinded or did not report blinding methods. Low intervention adherence and incomplete data was also a limitation of several RCTs. Uncertainty around whether participants represented the target population was a common issue for non-randomised designs, and the singular mixed methods study provided insufficient information regarding trustworthiness of the qualitative component, limiting comprehensive assessment.

**Table 2:** Summary of critical appraisal of the included studies using the Mixed Method Assessment Tool.

Study design	Criteria	Anderson [2017]	Behnammoghadam [2019]	Dearing & Kippenbrock [2022]	Ebrahimian [2021]	Gündoğan & Kaplan Serin [2022]	Hill [2021]	Jadidi [2023]	Mahdizadeh [2019]	Patterson [2015]	Patterson [2023a]	Patterson [2023b]	Patterson [2024]	Pinks [2021]	Porter & Johnson [2008]	Shabani [2023]	Shriane [2024]	Takeyama [2009]	Vaughan [2020]	Yoo [2023]
Randomised controlled trials	Is randomization appropriately performed?	▄	●		▄	▄		▄	●	●	●	●	●		▄	●	●			
	Are the groups comparable at baseline?	●	●		●	●		●	▄	●	●	▄	▄		▲	●	●			
	Are there complete outcome data?	●	●		●	●		●	▲	●	▲	●	●		▲	●	▲			
	Are outcome assessors blinded to the intervention provided?	▲	▄		▲	▲		●	▄	▲	●	▄*	▄*		▄	▲	▲			
	Did the participants adhere to the assigned intervention?	●	●		●	▄		▄	●	●	▲	●	●		▲	●	▲			
Quantitative non-randomised	Are the participants representative of the target population?			▄										●				▲	▄	●
	Are measurements appropriate regarding both the outcome and the intervention?			●										●				●	●	●
	Are there complete outcome data?			●										●				●	▲	●
	Are the confounders accounted for in the design and analysis?			▲										▄				●	●	●
	During the study period, is the intervention administered as intended?			●										●				●	▲	●
Mixed methods	Is there adequate rationale for using mixed methods design to address the research question?						●													
	Are the different components of the study effectively integrated to answer the research question?						●													
	Are the outputs of the integration of qualitative and quantitative components adequately interpreted?						●													
	Are divergences and inconsistencies between quantitative and qualitative results adequately addressed?						●													
	Do the different components of the study adhere to the quality criteria of each tradition of the methods involved?						▄													

Circle = Yes, Square = Unclear, Triangle = No. Grey boxes represent non-applicable item. *Marked ‘Can’t Tell’ based on information reported, however other publications on the same data set report statistician was blinded.

### Synthesis of results

#### Impact of psychological interventions.

Several studies reported a positive impact of a psychological intervention on indicators of mental well-being. *Eye Movement Desensitization and Reprocessing (EMDR)* showed a significant treatment effect in EMTs with average or higher stress levels. [62] However, since stress levels were ‘average’ both before and after the intervention, its efficacy for individuals with elevated stress remains unclear. A *PTSD prevention, surveillance, and education program* appeared to reduce PTSD symptoms in paramedics. [63] However, as the study did not report statistical significance testing, the reliability of this observed effect remains unclear. An *online resiliency training program* for paramedic students led to significant improvements in resilience scores. Yet, the timing of the intervention (i.e., prior to their first ambulance practicum) raises concerns about potential confounding from external influences. [61] The use of *‘psychological hotwash’* in EMS personnel resulted in a short-term increase in resilience, but this effect was not sustained at six-week follow-up, [64] while *peer support workshops* improved emotional expressivity and coping approaches in paramedic students. [65] However, there were no changes in depression and anxiety symptoms, or help-seeking behaviours in the latter study. This study, like the PTSD prevention study, was impacted by study design, and it is also unclear whether similar peer support workshops are beneficial in practicing paramedics. Finally, two interventions, *psychoeducation and group counselling*, [66] and a second *online resiliency training program,* [67] showed no significant effects on burnout, psychological symptoms, or resilience in small samples of paramedic students (*n* = 23 and *n* = 34, respectively).

These findings represent an early body of intervention literature, with a need for further robust investigations. Despite statistically significant findings in several of these studies, the clinical or practical significance is largely unclear beyond the modest sample sizes [62–65] and short follow-up timeframes. Specifically, there were no follow-up timepoints beyond post-intervention in three studies, limiting knowledge of any sustained treatment effects. Selection bias [65] and significant baseline between-group differences (age and BMI, [64] and sex [66]) were also evident. Outcome measures across studies were generally inconsistent, and while three studies examined resilience, each utilised a different measure of resilience making between-intervention comparisons challenging. Validity was also a concern relative to resilience scales used; little evidence is available on efficacy in individuals who live or work in challenging contexts, including the paramedic role. [80] Additionally, another study did not report evidence of scale validity for measuring stress [62].

#### Impact of sleep, fatigue and shift work interventions.

Five of the 19 studies examined sleep and fatigue interventions. Two studies reported moderate (*n* = 100 [68]) and large (*n* = 678 [70]) sample sizes, and another two utilised objective measures of sleep. [69,71] Two studies examined the impact of *napping* during night shifts on sleep, fatigue, mood, and cognitive performance, with somewhat contrasting outcomes. One study highlighted the benefit of longer naps, reporting that a 2-hour nap, compared to a 30-minute nap, significantly reduced sleepiness, fatigue, difficulty concentrating, and exhaustion in the six hours following a night shift. [69] Importantly, these naps did not impact subsequent sleep episodes, suggesting they are a viable strategy for improving recovery without compromising subsequent essential sleep opportunities. However, the second study recognised the drawbacks of napping mid-shift, with implications for translation. [71] Regardless of duration, napping led to increased sleep inertia and reduced alertness immediately upon waking. Additionally, the 2-hour nap increased sleepiness and reduced concentration right after waking relative to a 30-minute nap. Importantly, this study highlighted that these effects dissipated within 30 minutes, with no differences between nap lengths on measures of sleep, fatigue, or mood. These napping studies illustrate that while longer naps may support greater post-shift recovery, they may impair functioning immediately upon waking, which is problematic for paramedics who may need to respond urgently. Additionally, the feasibility of napping during shifts depends on operational demands and jurisdictional policies and will need to be considered in future studies.

Beyond napping, *sleep health and fatigue education*, [70] and *real-time text-messaging to support fatigue and sleep* [68] yielded differing findings. The education intervention had no effect on subjective reports of sleepiness, fatigue, and exhaustion, and sleep quality, which is perhaps unsurprising given that sleep health and hygiene information is often considered an active control in sleep interventions. The text-message intervention, however, improved sleep quality, while a *mobile sleep health application* [72] was suggestive of some improvement, albeit in a modest sample with significant study attrition. Observed effects in the text message study may be reflective of changes related to fatigue management whilst on shift. For example, improved sleep knowledge and awareness, together with encouraging on-shift naps, may have cumulatively supported better post-shift sleep. These findings are particularly important for high-demand and shiftwork settings, where low-burden interventions that facilitate behavioural change may offer a practical approach to improving sleep outcomes. While there were no between group (intervention vs control) effects in the mobile sleep health application on measures of insomnia, fatigue, sleepiness, and healthy sleep practices, within-subjects analyses suggested a reduction in insomnia symptom severity and behaviours which compromise sleep hygiene (e.g., engaging in wakeful activities before bed, going to bed stressed or angry). These findings suggest that the intervention may be beneficial, however, due to the pilot design, the study may have been underpowered to detect an effect.

It is important to consider the clinical importance relative to the findings of the text-message and mobile sleep health application studies. Despite observed differences in the text-message study post-intervention, suggesting an improvement in sleep quality, PSQI scores remained ≥5, suggestive of ongoing impairments to sleep quality. These findings may reflect the inherent limitations of sleep health education-focussed interventions within shift working populations. This is particularly the case when sleep disorders, which are prevalent in shift workers, [81,82] have not been considered. [83] The potential inclusion of participants with undiagnosed sleep disorders, who are less likely to respond to psychoeducational approaches in isolation, may have attenuated the intervention’s overall effectiveness. Similarly, the 2-point post-test reduction in insomnia severity in the mobile application study is below the 6-point reduction required to achieve clinical significance. High Insomnia Severity Index scores may have been due to high levels of fatigue prevalent in this population, or dissatisfaction with sleep schedules related to shift work, rather than the presence of insomnia. Additionally, as the authors state, a modest sample size and significant attrition may have also diminished the ability to quantify the impact of the intervention or, alternatively, reflect that the intervention is not meeting the needs of all shift workers recruited. Small sample sizes were also a concern for the napping studies, and low adherence was also present in the sleep health education study, possibly limiting intervention efficacy.

The singular study which targeted change at the organisational level, assessed a modified shift system which included scheduled, uninterrupted nap periods during night shift on subjective fatigue. [73] The authors reported that participants with scheduled sleep opportunities reported longer naps, however no significant differences were observed on subjective feelings of fatigue between traditional and modified shift types. This study had several limitations including a small sample (*n* = 10), a lack of validated measures for sleep and fatigue, and a quasi-experimental design.

#### Impact of complementary and alternative medicine interventions.

Five studies reported positive impacts of complementary and alternative medicine interventions on measures of sleep, fatigue and mental well-being. These included *foot reflexology and peppermint oil inhalation*, [78] *stachys lavandulifolia (anxiolytic tea)*, [76] *muscle relaxation training*, [74] *meditation*, [79] and use of a *wellbeing dog*. [75] Methodological limitations were common in these studies, and clinical importance of the effect of meditation or muscle relaxation training on anxiety and sleep, or meditation or anxiolytic tea on stress, was also uncertain. For anxiolytic tea use, inadequate information provided regarding the tool used to measure outcomes made it unclear whether the scores were clinically relevant. [76] The other two studies used samples with only mild anxiety or low levels of sleep difficulties at baseline, making it unclear whether muscle relaxation training or meditation would support improvements in the context of more severe symptoms. [74,79] All studies investigating an alternative therapy were limited by small sample sizes (*n* = 30 – *n* = 89), [74–79] three of which were non-representative due to a male-only sample. [76–78] Finally, the results of a study examining the effect of Swedish massage on occupational stress in EMTs were inconclusive due to outcome reporting discrepancies. [77]

## Discussion

This scoping review aimed to synthesise existing interventions for improving mental well-being and sleep in paramedics. Together, these studies highlight an interest in improving sleep and well-being, but a body of evidence which is impacted by small samples and highly variable outcome measurement scales utilized across the literature, which considerably impacted synthesis of findings. The largest number of studies (37%) came under the umbrella of ‘psychological’ interventions. Broadly, the existing literature specifically in paramedic samples provides limited evidence for potential psychological interventions to improve sleep or mental well-being outcomes. By contrast, there was early evidence that some strategies, such as napping on shift, may hold benefit, however the demanding paramedic work context and jurisdictional differences will need to be considered to address the feasibility of napping in practice. Complementary and alternative approaches offered poor quality of evidence overall at present. Taken together, findings of the included studies were limited by a range of factors including study design and quality, small or biased samples, no post-intervention follow-up timepoints, a lack of comparable interventions and outcomes, and low psychological symptoms at baseline. The majority of reported interventions focussed on the individual, rather than on addressing systemic contributors to challenges with sleep and mental health – such as organisational structure and support, shift schedules, and workload.

### Effectiveness and engagement of interventions

Although the majority of studies included in this review provided limited or preliminary evidence regarding the effectiveness of interventions, they nonetheless offer valuable contributions to the literature. Some studies represented pilot-type investigations, offering useful insights into feasibility, acceptability, and potential implementation challenges in the context of paramedicine. For example, Shriane et al reported low response rates and high attrition for their *Sleepfit* intervention, which may have reflected broader systemic barriers to participation (e.g., shift work, limited sleep hygiene knowledge, low help seeking). [72] This is important insight for future intervention development. Future research efforts could therefore address these barriers through leveraging co-design approaches and sleep health education. Similarly, Patterson et al also reported low engagement in their sleep health education intervention, despite participants reporting acceptability of educational content. [70] These findings highlight that building sleep health education interventions into existing organisational structures may be an important approach, potentially addressing systemic barriers to engagement and aligning with broader findings of the benefits from organisational interventions in shift workers. [84] Shriane et al and Patterson et al also identified low engagement with the digital delivery format itself, suggesting that e-based interventions, while convenient for some populations, may not be well-suited to this cohort without additional support or integration considerations. [85] This further underscores the need for interventions that are not only contextually relevant, but also accessible and engaging within the realities of shift work environments. Moreover, the collective body of evidence of the included studies underscores the urgent need for more rigorous and longitudinal research in paramedic wellbeing, ideally informed by the end-user. [86] By highlighting critical gaps, such as a lack of standardized outcome measures, limited focus on sleep-specific interventions, and minimal consideration of organisational factors, these studies provide a roadmap for shaping future investigations that are more robust, targeted, and impactful.

Substantial heterogeneity was evident in terms of both interventions applied and outcome measures used. Studies examining sleep interventions tended to use consistent measures (two including objective sleep measures), and validated questionnaires were generally utilised across studies. A challenge in the existing literature is determining whether any observed changes in sleep or mental health are sustained in the medium- or long-term, as there is a paucity of literature which included multiple follow-ups. The clinical relevance of interventions was also not well articulated in the existing literature. For example, despite reporting statistically significant change, three studies reported mild or ‘average’ levels of mental health symptomology both pre- and post-intervention. [62,74,79] It is important to note that the same intervention delivered to individuals experiencing high levels of symptoms may have a different effect, which needs to be better explored in further studies given the high rates of clinically significant anxiety and depression in this workforce. [6,11] Similarly, despite significantly improved sleep quality post-intervention, participants in another study still reported significant post-test sleep difficulties. [68] Consequently, while statistically significant effects are observed, this does not necessarily constitute clinically meaningful differences in symptoms, nor translate to potential benefit for those with different symptom profiles.

Resilience was a common factor among components of the interventions as well as outcome measures, with low quality evidence to support interventions aimed at resilience building. While developing individual resilience seems logical and is commonly examined in first responder cohorts, [87] relying on individual adaptation to challenging work conditions may not be the best approach in isolation. Taking a systems approach has been highlighted as fundamental in efforts to support worker mental well-being broadly, where integrated solutions targeted at all levels of the system are required. [88] A systems approach can be operationalised as primary (working conditions), secondary (short term responses to stress), and tertiary (long term illness intervention) solutions. The current review suggests that paramedic mental well-being and sleep is not being addressed systemically. More broadly, reviews highlight limited system-level interventions to support mental well-being in healthcare workers beyond paramedicine, and clarify the need for such approaches. [89,90] Additionally, the efficacy of resilience building interventions in psychologically hazardous environments remains uncertain. [80] Given no universal definition of resilience, it may also be useful to consider what this truly means in the paramedic context.

### Systemic considerations and future directions

When considering meaningful interventions, known contributors to poor mental well-being and sleep in paramedics are an important consideration. A key finding of this review is the disconnect between individual-focused interventions, and the broader systemic nature of stressors for paramedics [91,92]. Poor work systems and chronic occupational stressors are increasingly recognised in paramedics, suggesting that well-designed interventions targeting organisational systems may be required. This is consistent with findings from the 2021 *SWAP* report, which identified that wellbeing support across UK ambulance services were fragmented and primarily individual-focused, with few integrated, system-level initiatives. [93] Yet, only one study identified in this review focussed on organisation-level change. Further, qualitative research has identified a range of barriers to paramedics accessing mental health supports, [94] and help-seeking behaviours among paramedics are low. [18] Interventions which are embedded within paramedic operations and education systems may be beneficial. For example, Brown et al [95] found that screening and management of sleep disorders was both feasible and acceptable in Australian paramedic students, and may hold benefit for improving sleep before the transition into paramedic careers, particularly given that early paramedic careers are associated with changes to health behaviours, including sleep quality. [96] Finally, proactive approaches to paramedic mental well-being were limited in this review. While reactive strategies (e.g., peer support programs) play an important role for reactive approaches to trauma exposure, [97] focus also needs to be drawn toward strategies which mitigate the impact of both trauma exposure and chronic occupational stressors, and prioritise early identification of mental health and sleep problems.

Limited evidence found in this review is likely reflective of an evolving profession. [2] Efforts made to advance evidence in this area over the last decade indicate recognition of the importance of paramedic well-being and, importantly, highlight an opportunity for development. A critical component of this endeavour is collaboration. The evolving nature of paramedicine research, and the subsequent impact on research quality within this discipline, has been discussed. [98] To support quality, research in paramedicine can be bolstered through collaboration within and beyond the profession, which may facilitate broader perspectives, knowledge integration and improved research design. [98] The benefit of interdisciplinary collaboration is evident in this review, where studies reporting higher quality research, were primarily from multidisciplinary research groups, and well-established fields of research. Collaboration also includes the participation of both paramedics and relevant organisations, and stakeholders in the scientific process. To achieve this, it is essential that paramedics themselves are supported to develop education and skills necessary to engage in research. Currently, however, paramedic academics are a vastly underrepresented group and require greater support to grow as an academic profession. [99]

### Limitations

The present review should be considered in light of some limitations. Several studies were identified in the screening process which included paramedics among several other first responder groups (e.g., police, firefighters) in an intervention study. However, commonly, outcomes were not reported by occupation. Therefore, there is likely more evidence regarding interventions for paramedic mental well-being and sleep, but we believe there are important differences between first responder groups which may confound outcomes in studies of broader groups. Further, our focus on peer-reviewed sources which empirically evaluated an intervention meant that unpublished organisational programs or initiatives were not included. We acknowledge that these may represent valuable approaches to supporting paramedic mental well-being and sleep and could be evaluated in future research to further develop the evidence-base for this work group. Additionally, non-English articles, as well as theses and dissertations were excluded from this review. While this decision was made to prioritise consistency in study appraisal and methodological reporting, we acknowledge that relevant evidence may not have been captured, possibly limiting the breadth of available findings.

The lack of a universal role title for paramedics posed a challenge for this review. For example, ambulances in the Netherlands are staffed by nurses and in Iran by nurses, nurse aides, and ‘technicians’. Although we included studies where these workers were classified as ‘emergency medical service workers’, it was necessary to exclude studies examining nurses in ambulance settings, as nurses and other occupation data were explicitly excluded in several of the included studies. Developing a universally accepted role title has been identified as an essential factor in the development of the discipline of paramedicine broadly, [100] and is likely a key component in the advancement of evidence surrounding paramedic well-being. Additionally, while many studies have examined interventions for broader first responder groups, paramedics are typically a small proportion of the sample, and/or data is not able to be independently interpreted by occupation. It is important that future research efforts target paramedics as an individual profession who have unique stressors and barriers related to their roles.

## Conclusions

Poor mental well-being and suboptimal sleep are common among paramedics, yet the body of literature on targeted interventions to address these issues remains limited. Most available studies offer preliminary or low-quality evidence, reflecting both the evolving nature of the research area and the relative novelty of paramedicine as a profession. Existing interventions have largely focussed on individual-level change (e.g., resilience, psychoeducation), while overlooking broader systemic contributors to poor mental well-being and sleep. Additionally, evidence for longer term impact or clinical importance is limited, and real-world applicability of some interventions is uncertain. Although some sleep-focussed interventions were identified, studies were seldom designed to explicitly improve mental well-being. Issues such as low adherence and engagement point to systemic barriers such as shift work and low help-seeking behaviours. Addressing these challenges requires system-level strategies targeting working conditions, policy, education, and embedded support mechanisms. Interdisciplinary collaboration, inclusion of paramedics in research design, and greater support for paramedic-led research are critical to developing effective, contextually relevant interventions to support this essential workforce.

## Supporting information

S1 Table Summary of searches. (DOCX)

S2 Table Data charting plan. (DOCX)

S3 Table Outcome measures utilised in the included studies. ^a^Validated scale, ^b^Objective measure.
(DOCX)

S4 Table PRISMA-ScR Checklist. (DOCX)
