# Supplementary material for: Interventions to improve mental well-being and sleep in paramedics: A scoping review
Source: PLoS One. 2026 Mar 9;21(3):e0344377. doi: 10.1371/journal.pone.0344377 (PMC12970977; doi:10.1371/journal.pone.0344377)
Supplement: S1 Table — (DOCX) [file pone.0344377.s001.docx]

**S1 Table. Summary of searches**

| **Date of initial search** | **Database** | **Records retrieved/screened** | **Date of revised strategy search** | **Records retrieved/screened** |
| --- | --- | --- | --- | --- |
| 1 Feb 24 | CINAHL | 433 | 28 Oct 24 | 515 |
| 1 Feb 24 | Web of Science | 2856 | 28 Oct 24 | 384 |
| 1 Feb 24 | MEDLINE | 938 | 28 Oct 24 | 743 |
| 2 Feb 24 | PsycINFO | 244 | 28 Oct 24 | 313 |
| 21 Feb 24 | Google Scholar | 145 | N/A | N/A |
| 21 Feb 24 | Amber | 368 | N/A | N/A |
| 21 Feb 24 | Trove | 1423 | N/A | N/A |
| **CINAHL search string**  ((MH Paramedicine OR MH Paramedics OR MH "Emergency Medical Technicians" OR MH "Emergency Responders" OR MH "Emergency Medical Services" OR MH Ambulances) OR (TI paramedic* OR TI "emergency medical technician*" OR TI "first responder*" OR TI "emergency responder*" OR TI "emergency medical service*" OR TI ambulance* OR TI "ambulance personnel" OR TI "emergency service*" OR TI "emergency service personnel" OR TI "public safety personnel" OR TI "helicopter emergency medical service*" OR TI HEMS OR AB paramedic* OR AB "emergency medical technician*" OR AB "first responder*" OR AB "emergency responder*" OR AB "emergency medical service*" OR AB ambulance* OR AB "ambulance personnel" OR AB "emergency service*" OR AB "emergency service personnel" OR AB "public safety personnel" OR AB "helicopter emergency medical service*" OR AB HEMS)) AND ((MH "Clinical Trials" OR MH Psychotherapy) OR (TI trial OR TI program* OR TI intervention OR TI therapy OR AB trial OR AB program* OR AB intervention OR AB therapy)) AND ((MH Fatigue OR MH "Quality of Life" OR MH "Mental Health" OR MH "Posttraumatic Growth" OR MH Emotions OR MH Anger OR MH Anxiety OR MH Depression OR MH "Emotional Regulation" OR MH "Psychological Distress" OR MH "Psychological Trauma" OR MH "Post Traumatic Stress Disorder" OR MH "Acute Stress Disorder" OR MH "Burnout, Psychological" OR MH "Occupational Stress" OR MH Sleep OR MH "Sleep Hygiene" OR MH "Sleep Duration" OR MH "Resilience, Psychological") OR (TI fatigue OR TI "quality of life" OR TI “well being” OR TI wellbeing OR TI "mental health" OR TI "post traumatic growth" OR TI emotion* OR TI psycholog* OR TI anger OR TI anxi* OR TI depress* OR TI "emotional regulation" OR TI "psychological distress" OR TI stress* OR TI PTSD OR TI "post traumatic stress disorder" OR TI burnout OR TI sleep* OR TI insomnia OR TI coping OR TI resilien* OR TI wellness OR AB fatigue OR AB "quality of life" OR AB “well being” OR AB wellbeing OR AB "mental health" OR AB "post traumatic growth" OR AB emotion* OR AB psycholog* OR AB anger OR AB anxi* OR AB depress* OR AB "emotional regulation" OR AB "psychological distress" OR AB stress* OR AB PTSD OR AB "post traumatic stress disorder" OR AB burnout OR AB sleep* OR AB insomnia OR AB coping OR AB resilien* OR AB wellness)) AND (PY 2004-2024) | | | | |
| **Web of Science search string**  (TI=(paramedic* OR "emergency medical technician*" OR "first responder*" OR "emergency responder*" OR "emergency medical service*" OR ambulance* OR "ambulance personnel" OR "emergency service*" OR "emergency service personnel" OR "public safety personnel" OR "helicopter emergency medical service*" OR HEMS) OR AB=(paramedic* OR "emergency medical technician*" OR "first responder*" OR "emergency responder*" OR "emergency medical service*" OR ambulance* OR "ambulance personnel" OR "emergency service*" OR "emergency service personnel" OR "public safety personnel" OR "helicopter emergency medical service*" OR HEMS) OR AK=(paramedic* OR "emergency medical technician*" OR "first responder*" OR "emergency responder*" OR "emergency medical service*" OR ambulance* OR "ambulance personnel" OR "emergency service*" OR "emergency service personnel" OR "public safety personnel" OR "helicopter emergency medical service*" OR HEMS)) AND (TI=(trial OR program* OR intervention OR therapy) OR AB=(trial OR program* OR intervention OR therapy) OR AK=(trial OR program* OR intervention OR therapy)) AND (TI=(fatigue OR "quality of life" OR “well being” OR wellbeing OR "mental health" OR "post traumatic growth" OR emotion* OR psycholog* OR anger OR anxi* OR depress* OR "emotional regulation" OR "psychological distress" OR stress* OR PTSD OR "post traumatic stress disorder" OR burnout OR sleep* OR insomnia OR coping OR resilien* OR wellness) OR AB=(fatigue OR "quality of life" OR “well being” OR wellbeing OR "mental health" OR "post traumatic growth" OR emotion* OR psycholog* OR anger OR anxi* OR depress* OR "emotional regulation" OR "psychological distress" OR stress* OR PTSD OR "post traumatic stress disorder" OR burnout OR sleep* OR insomnia OR coping OR resilien* OR wellness) OR AK=(fatigue OR "quality of life" OR “well being” OR wellbeing OR "mental health" OR "post traumatic growth" OR emotion* OR psycholog* OR anger OR anxi* OR depress* OR "emotional regulation" OR "psychological distress" OR stress* OR PTSD OR "post traumatic stress disorder" OR burnout OR sleep* OR insomnia OR coping OR resilien* OR wellness)) AND PY=(2004-2024) | | | | |
| **PsycINFO search string**  APA PsycInfo <1806 to October 2024 Week 4>  1 (paramedicine or paramedics or emergency medical technicians or emergency responders or emergency medical services or ambulances+).sh. 537  2 (paramedic* or emergency medical technician* or emt or first responder* or emergency responder* or emergency medical service* or ems or ambulance* or ambulance personnel or emergency service* or emergency service personnel or public safety personnel or psp or helicopter emergency medical service* or hems).tw. 9999  3 1 or 2 10018  4 (paramedic* or emergency medical technician* or first responder* or emergency responder* or emergency medical service* or ambulance* or ambulance personnel or emergency service* or emergency service personnel or public safety personnel or helicopter emergency medical service* or hems).tw. 7140  5 1 or 4 7162  6 (clinical trial+ or psychotherapy+).sh. 61244  7 (trial or program* or intervention or therapy).tw. 1047053  8 6 or 7 1078061  9 5 and 8 2147  10 (fatigue+ or quality of life+ or mental health or posttraumatic growth, psychological or emotions or anger or anxiety or depression or emotional regulation or psychological distress, psychological or psychological trauma or stress disorder, post-traumatic or stress disorders, traumatic, acute or burnout, psychological+ or occupational stress or sleep or sleep hygiene+ or sleep duration or resilience, psychological).sh. 390417  11 (fatigue or quality of life or well-being or wellbeing or well being or mental health or post#traumatic growth or emotion* or psycholog* or anger or anxi* or depress* or emotional regulation or psychological distress or stress* or post traumatic stress disorder or ptsd or burnout or sleep* or insomnia or coping or resilien* or wellness).tw. 1923765  12 10 or 11 1938683  13 9 and 12 1199  14 limit 13 to yr="2004 -Current" 1005 | | | | |
| **MEDLINE search string**  1 (paramedicine or paramedics or emergency medical technicians or emergency responders or emergency medical services or ambulances+).sh. 59367  2 (paramedic* or emergency medical technician* or emt or first responder* or emergency responder* or emergency medical service* or ems or ambulance* or ambulance personnel or emergency service* or emergency service personnel or public safety personnel or psp or helicopter emergency medical service* or hems).tw,kf. 101635  3 1 or 2 139594  4 (paramedic* or emergency medical technician* or first responder* or emergency responder* or emergency medical service* or ambulance* or ambulance personnel or emergency service* or emergency service personnel or public safety personnel or helicopter emergency medical service* or hems).tw,kf. 46636  5 1 or 4 87153  6 (clinical trial+ or psychotherapy+).sh. 598463  7 (trial or program* or intervention or therapy).tw,kf. 4703903  8 6 or 7 5066434  9 5 and 8 17590  10 (fatigue+ or quality of life+ or mental health or posttraumatic growth, psychological or emotions or anger or anxiety or depression or emotional regulation or psychological distress, psychological or psychological trauma or stress disorder, post-traumatic or stress disorders, traumatic, acute or burnout, psychological+ or occupational stress or sleep or sleep hygiene+ or sleep duration or resilience, psychological).sh. 757135  11 (fatigue or quality of life or well-being or wellbeing or well being or mental health or post#traumatic growth or emotion* or psycholog* or anger or anxi* or depress* or emotional regulation or psychological distress or stress* or post traumatic stress disorder or ptsd or burnout or sleep* or insomnia or coping or resilien* or wellness).tw,kf. 3133628  12 10 or 11 3247725  13 9 and 12 2398  14 limit 13 to yr="2004 -Current" 1964 | | | | |
| **Google Scholar search string**  Paramedic \| “emergency medical technician” \| “ambulance personnel” \| “emergency service personnel” \| “first responder” intervention \| trial \| program mental \| psychological \| wellbeing \| well-being \| PTSD \| anxiety \| depression \| burnout \| stress \| resilience \| coping \| sleep \| fatigue | | | | |
| **Amber search string**  (paramedic OR “emergency medical technician” OR “ambulance personnel” OR “emergency service personnel” OR “first responder”) AND (intervention OR trial OR program) AND (mental OR psychological OR wellbeing OR well-being OR ptsd OR anxiety OR depression OR burnout OR stress OR resilience OR coping OR sleep OR fatigue) | | | | |
| **Trove search string**  (paramedic OR “emergency medical technician” OR “ambulance personnel” OR “emergency service personnel” OR “first responder”) AND (intervention OR trial OR program) AND (mental OR psychological OR wellbeing OR well-being OR ptsd OR anxiety OR depression OR burnout OR stress OR resilience OR coping OR sleep OR fatigue) | | | | |
