# Supplementary material for: Interventions to improve mental well-being and sleep in paramedics: A scoping review
Source: PLoS One. 2026 Mar 9;21(3):e0344377. doi: 10.1371/journal.pone.0344377 (PMC12970977; doi:10.1371/journal.pone.0344377)
Supplement: S2 Table — (DOCX) [file pone.0344377.s002.docx]

**S2 Table. Data charting plan**

| **Data items**   - Author/s - Year of publication - Publication title - Study location (country) and who is conducting the study (e.g., charity, organisation, university) - Source type (e.g., journal article, thesis) and location (e.g., grey literature) - Sample size (only for paramedic population if other occupations also investigated) - Population specifics (age, gender/sex, occupational role title, other relevant occupation specifics [e.g., employer/workplace type, years of service, shift work status], relevant mental or sleep health information [e.g., paramedics diagnosed with PTSD]) - Aims of study - Methods overview (study design, theoretical frameworks) - Intervention (what [mental-wellbeing, sleep] and how was it delivered [e.g., online, in-person], when was it delivered [preventative or reactive], where was it delivered [e.g., community, organisation], who was it delivered by) - Outcome measures (what measures evaluated, validated or non-validated) - Results (summary including efficacy) - Limitations - Synthesis/summary of evidence will broadly consider a) Areas of mental well-being that have been targeted for intervention b) what interventions have been considered and have sleep interventions been considered? c) how and in what setting /context are the interventions delivered? d) who is delivering the intervention? e) when is the intervention delivered? |
| --- |
| **Assumptions and simplifications**   - Where necessary, corresponding authors will be contacted for any missing data required to conduct the scoping review. |
