# Supplementary material for: Interventions to improve mental well-being and sleep in paramedics: A scoping review
Source: PLoS One. 2026 Mar 9;21(3):e0344377. doi: 10.1371/journal.pone.0344377 (PMC12970977; doi:10.1371/journal.pone.0344377)
Supplement: S3 Table — aValidated scale, bObjective measure. (DOCX) [file pone.0344377.s003.docx]

**S3 Table. Outcome measures utilised in the included studies**

| **Outcome type** | **Outcome measure** | **Frequency (n)** |
| --- | --- | --- |
| Sleep | Pittsburgh Sleep Quality Index (PSQI)^a^ | 4 |
|  | Epworth Sleepiness Scale (ESS)^a^ | 3 |
|  | Electroencephalogram (EEG)^a,b^ | 2 |
|  | Self-reported sleepiness, fatigue, and mood | 2 |
|  | Insomnia Severity Scale (ISI)^a^ | 1 |
|  | Leeds Sleep Evaluation Questionnaire (LSEQ)^a^ | 1 |
|  | Psychomotor Vigilance Test (PVT-B)^a,b^ | 1 |
|  | Self-reported nap timing | 1 |
|  | Sleep diary | 1 |
|  | Sleep Hygiene Index (SHI)^a^ | 1 |
|  | Wrist actigraphy^a,b^ | 1 |
| Depression, anxiety, and stress | Beck Anxiety Inventory (BAI)^a^ | 1 |
|  | Depression Anxiety and Stress Scale (DASS-21)^a^ | 1 |
|  | Elkin/Alken Stress Symptom Scale | 1 |
|  | Expanded Nurses Stress Scale (ENSS) | 1 |
|  | Generalized Anxiety Disorder questionnaire (GAD-7)^a^ | 1 |
|  | Hospital Stress Scale (HSS-35) | 1 |
|  | Occupational Stress Inventory (OSIPOW)^a^ | 1 |
|  | PTSD checklist for DSM-5 (PCL-5)^a^ | 1 |
|  | Stress Response Inventory (SRI)^a^ | 1 |
|  | Symptom Checklist-90-Revised (SCL-90-R)^a^ | 1 |
|  | The Center for Epidemiological Studies Depression Scale - Korean version (K-CES-D)^a^ | 1 |
| Resilience, coping, and burnout | Emergency Medical Services Resilience Scale (EMSRS)^a^ | 1 |
|  | Emotional Approach Coping Scale (EACS)^a^ | 1 |
|  | Maslach Burnout Inventory (MBI)^a^ | 1 |
|  | Multidimensional Scale of Perceived Social Support (MPSS)^a^ | 1 |
|  | Peer support crisis questionnaire | 1 |
|  | Resilience Scale (RS)^a^ | 1 |
|  | Resilience Scale for Adults (RSA)^a^ | 1 |
|  | Ways of Coping (WOC)^a^ | 1 |
| Attitudes and behaviours | Schedule Attitudes Survey (SAS)^a^ | 2 |
|  | Attitudes Toward Emotional Expression scale (AEE)^a^ | 1 |
|  | EMS Safety Attitudes Questionnaire (EMS-SAQ)^a^ | 1 |
|  | General Help Seeking Questionnaire (GHSQ)^a^ | 1 |
|  | Sleepiness Fatigue & Alertness Behaviour Survey (SFAB) | 1 |
| Emotion and quality of life | Brief Emotional Experience Scale (BEES)^a^ | 1 |
|  | Emotional Expressivity Scale (EES)^a^ | 1 |
|  | Fear of COVID-19 Scale (CP19-S)^a^ | 1 |
|  | Professional Quality of Life (ProQOL)^a^ | 1 |
| Fatigue | Chalder Fatigue Questionnaire (CFQ)^a^ | 2 |
|  | Fatigue Severity Scale (FSS)^a^ | 2 |
|  | Occupational Fatigue Exhaustion Recovery scale (OFER)^a^ | 2 |
|  | Subjective fatigue questionnaire | 1 |

^a^Validated scale, ^b^Objective measure.
